# Antidoting Crude Drugs with Potencies

**Published:** 1894-07

**Authors:** A. W. Holcombe

**Affiliations:** Kokomo, Ind.


### ANTIDOTING CRUDE DRUGS WITH POTENCIES.

A. W. Holcombe, M. D., Kokomo, Ind.

Editor Homœopathic Physician :—The antidotal relations between crude drugs and their potentized form, or the treatment of artificial diseases, is occupying more or less of the attention of the profession just now. Some believe, some doubt, some ridicule the idea of the high potency of a drug, antidoting the effects of the crude drug. A *few* there be who have put it to the *test* as Hahnemann directs, and the results have shown that it is *true*, that the law of *Similia* is universal and infallible. I enclose reports of three cases, taken from among *many* similar ones.

Mrs. G., æt. fifty, brunette. Eczema on hands, in palms, and on dorsum. Dry, cracked, burns and itches, worse at night, and after putting hands in water. Brother and sister died of consumption. Has hot flashes passing off with sweat—soles of feet burn, and has a red eruption on soles. Can't stand heat, of stove or bed. Has stomach trouble, can't eat cabbage. Took large quantities of Sarsaparilla and Iodide of Potash some years ago for the blood. Also, *lots* of Quinine. Had had Sulphur, but it didn't cure her. Believing that the drug or artificial disease from the Kali-iod. prevented the other remedies from acting, I gave her a dose of Kali-iod.$^{dmm}$ (Swan), and in less than ten days the eczema was entirely healed and skin smooth.

Mrs. S., æt. about sixty-five. Has symptoms of a cold. Cough worse at night, not much expectoration. Hoarse, rawness and soreness of throat—thirsty; drinking relieves the throat. Sweats at night. No appetite for breakfast, but gets hungry during forenoon. Pain in right chest on coughing, feet hot, also hands. Soles of feet feel as if they were squeezed. Constipated, urine has red sediment, urine passed frequently during night, or as soon as she gets on the feet. She had been under homœopathic treatment for months before I saw her, but the remedies only seemed to palliate her condition Upon inquiry I found she had worn artificial teeth, with *red rubber* plate for twenty years. Knowing that *red* rubber plate in artificial teeth contains a large proportion of free Mercury, I judged that the system had absorbed enough of it to establish an *artificial* disease. I gave her a powder of Merc-viv.$^{dmm}$ (Swan). In about ten days an eruption came out all over the palms of the hands, similar to small blood-blisters, though not quite so dark. This disappeared after several days and she improved gradually, and when improvement stopped she was given a dose of Sulphur$^{3mm}$, which completed the work.

Mrs. P., æt. fifty-four. Had a similar experience as the case just mentioned. Feels so weak and nervous—violent palpitation of heart at times, with shortness of breath; worse when lying down and after eating. Sharp pains through shoulders into chest. Dyspnœa, worse from exertion, in evening and at night. So weak in limbs. Feet burn and sweat. Pain from nape of neck into head. Constipated. Urinates too often at night. Thirsty of mornings. Don't like cold air to blow on her, it aggravates the pain in neck. Whitish, sandy sediment in urine. Limbs swollen, worse at night, limbs feel heavy, stiffness of knees, tingling in limbs. Has worn artificial teeth with *red rubber plate* for sixteen years. Has been under good homœopathic treatment for months with no permanent benefit. Evidently the Merc. impression prevented the action of the other remedies. Gave Merc-viv.$^{dmm}$ (Swan). Reported in two weeks better in *every* way, better than for many months. Placebo. Case went on improving with no other medicine and she now declares herself well. A case is always difficult to treat when the patient has *red rubber* plate or several *amalgam* fillings in the teeth. I always order the fillings removed and the plate changed for a black rubber one, and then antidote the Mercury with Merc-viv. *high*. After doing this I have frequently seen one prescription do more in a month's time than had before been accomplished in a year.

